# Has Japan overcome COVID-19 pandemic-associated frailty by 2024? Third report

**DOI:** 10.1016/j.jnha.2025.100495

**Published:** 2025-01-27

**Authors:** Tamaki Hirose, Yohei Sawaya, Masahiro Ishizaka, Naori Hashimoto, Akira Kubo, Tomohiko Urano

**Affiliations:** a Department of Physical Therapy, School of Health Sciences, International University of Health and Welfare, Otawara, Tochigi, Japan; b Senior Services Division of Otawara, Otawara, Tochigi, Japan; c Department of Physical Therapy, School of Health Sciences at Odawara, International University of Health and Welfare, Odawara, Kanagawa, Japan; d Department of Geriatric Medicine, School of Medicine, International University of Health and Welfare, Narita, Chiba, Japan

As of December 2024, approximately 5 years have passed since coronavirus disease 2019 (COVID-19) spread in Japan. Reflecting on the trajectory thus far, the period from 2020 to 2021 saw significant concerns about reduced activity levels among older adults owing to prolonged self-isolation, which had a profound impact [1]. Subsequently, vaccination efforts began in May 2021, and from 2022 onward, there was a trend toward easing behavioral restrictions. Furthermore, in May 2023, the Japanese government reclassified COVID-19 under the Infectious Diseases Control Law, changing its legal status from Class 2 to Class 5. In Japan, the prevalence of frailty increased in 2020 and 2021 following the onset of the COVID-19 pandemic. During this period, the heightened rates of frailty and pre-frailty were recognized as a concern and referred to as “pandemic-associated frailty” [2]. Signs of recovery began to emerge in 2022, and by 2023, frailty prevalence appeared to approach pre-pandemic levels in Japan [3]. Changes in physical and social activities were reported to have fully returned to pre-COVID-19 pandemic levels by 2023 [4]. However, there is no evidence to confirm whether the impact of “pandemic-associated frailty” has been wholly mitigated or whether the prevalence of frailty has fully reverted to pre-pandemic levels. This study, conducted with the latest data added, aimed to clarify changes in frailty among community-dwelling older adults in Japan over 8 years from 2017 to 2024. This study examined whether Japan has fully overcome “pandemic-associated frailty.”

This is a repeated cross-sectional study. Between 2017 and 2024, an annual survey was conducted in May or June targeting all 70- and 75-year-old residents of City A in Tochigi Prefecture, excluding those certified as requiring long-term care. The survey utilized the Kihon Checklist (KCL) via a mailed questionnaire, with the periods divided as follows: 2017–2021 (First Report) [2], 2022–2023 (Second Report) [3], and 2024 (present survey). The dataset for 2024, comprising 973 participants, was added for the analysis after excluding non-respondents, individuals who declined to participate, those undergoing medical treatment, and those with missing data. Frailty was assessed using the Kihon Checklist (KCL) based on prior research, with total scores categorized as follows: 0–3 points indicating robustness, 4–7 points as pre-frailty, and 8 points or more indicating frailty [1,2]. This study was approved by the Ethics Committee of the International University of Health and Welfare (21-Io-38-2, 22-Io-25), and participant consent was obtained using an opt-out approach. The study adhered to the principles of the Declaration of Helsinki. Statistical analyses included examining frailty status transitions over 8 years and calculating the proportion of responses scoring 1 point for KCL Questions 4 (interaction with friends) and 17 (going out).

The frailty status in 2024 was as follows: robust, 58.0%; pre-frailty, 29.2%; and frailty, 12.8% (Fig. 1). Figure S1 illustrates the proportion of individuals who scored 1 point for Question 4, “Do you sometimes visit your friends?” (answering “No”), and question No. 17, “Do you go out less frequently compared to last year?” (answering “Yes”). In 2024, the proportion for each item was 22.9% for Question 4 (interaction with friends) and 12.1% for Question 17 (going out).

**Fig. 1:**
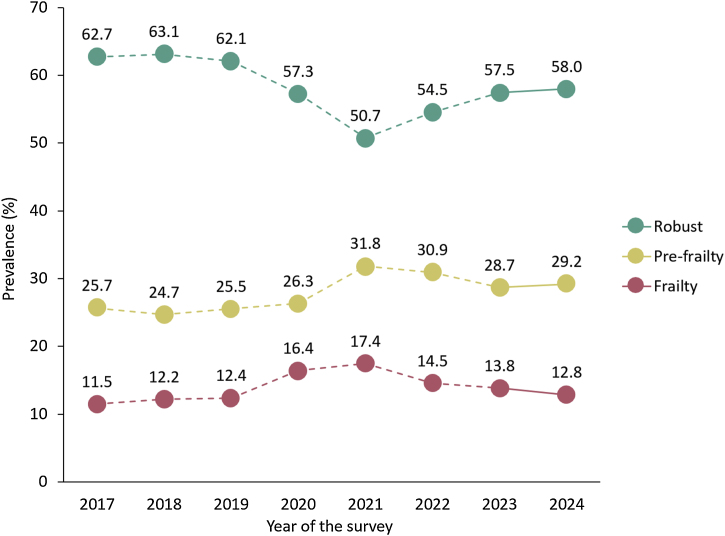
Changes in frailty prevalence over 8 years. By incorporating the 2024 analysis into our previously reported data, we presented the changes in the prevalence of frailty over 8 years. In 2024, the prevalence of frailty returned to levels observed before the COVID-19 pandemic.

The 2024 results revealed two distinct characteristics of pandemic-associated frailty. First, by 2024, the prevalence of frailty and the proportion of individuals responding affirmatively to Question 17 (going out less frequently) had fully recovered, reaching the levels observed before the COVID-19 pandemic. This indicates that while frailty experienced a significant surge during the pandemic, it has returned to pre-pandemic levels. Second, the prevalence of pre-frailty and the proportion of individuals with fewer interactions with friends (as indicated by Question 4) did not fully return to pre-COVID-19 pandemic levels. Improvement from frailty to pre-frailty has generally been reported in a review article, but reports of a two-step direct improvement from frailty to robust condition are somewhat uncommon [5]. In practice, taking the COVID-19 pandemic period as an example, the physical activity time of individuals with frailty was only approximately 40% of that of robust individuals [6]. Considering previous findings and this study’s results, pre-frailty may not have returned to normal post-COVID-19 pandemic because a “return to normal” takes time. In other words, we can speculate that a fraction of people reverted from frailty to pre-frailty over 3–4 years after the pandemic and that they are “on the path” to robust condition in the coming years. In terms of going out, possibly, smaller-scale activities, such as outings alone or with family, have recovered. However, larger community-based activities may still be affected by the lingering impact of pandemic-associated frailty. These findings indicate that while pandemic-associated frailty has generally shown signs of resolution, the key areas requiring attention and intervention in older adults are “pre-frailty” and “social interaction.” Pre-frailty is an early, reversible risk state before frailty develops. Even when there are no apparent functional issues, this condition is more prone to deterioration and transition to frailty in the future. These results indicate that timely strategies are required for early detection and proper intervention for pre-frailty. Furthermore, it is important to address the decline in social interactions, such as reduced interactions with friends and lifestyle habits of individuals living alone, which may not be easily noticed by others [7,8].

Its focus on a single city and two age groups limited this study. However, it provides significant insights into whether Japan has overcome pandemic-associated frailty, a topic that has not been sufficiently addressed. Following the COVID-19 pandemic, a report showed the number of individuals requiring long-term care certification has increased [9]. Although they may not need long-term care certification, the number of individuals in a potentially frail state, similar to pre-frailty, may be increasing. Continuous follow-ups for approximately 10 years are required.

## CRediT authorship contribution statement

Conceptualization: TH, YS, TU. Data curation: TH, YS, MI, NH. Formal analysis: YS. Funding acquisition: TH, YS, TU. Investigation: TH, YS, MI, NH, AK. Methodology: TH, YS, MI, TU. Project administration: MI, NH, AK. Resources: MI, NH. Software: YS. Supervision: AK, TU. Validation: TH, YS. Visualization: YS. Writing-original draft: TH, YS. TU. Writing-review & editing: All.

## Sponsor’s role

The funders had no role in the design, methods, participant recruitment, data collection, analysis, and manuscript preparation.

## Funding information

This study was funded by JSPS Grants-in-Aid for Scientific Research (22K11096, 22K17539 and 23K06873) and the JGS Grant for Geriatric Nutrition Research supported by Otsuka Pharmaceutical Factory, Inc.

## Declaration of competing interest

The authors declare that there is no conflict of interest.
