# Supplementary material for: Has Japan overcome COVID-19 pandemic-associated frailty by 2024? Third report
Source: J Nutr Health Aging. 2025 Jan 27;29(4):100495. doi: 10.1016/j.jnha.2025.100495 (PMC12180047; doi:10.1016/j.jnha.2025.100495)
Supplement: Supplementary file 1 [file mmc1.docx]

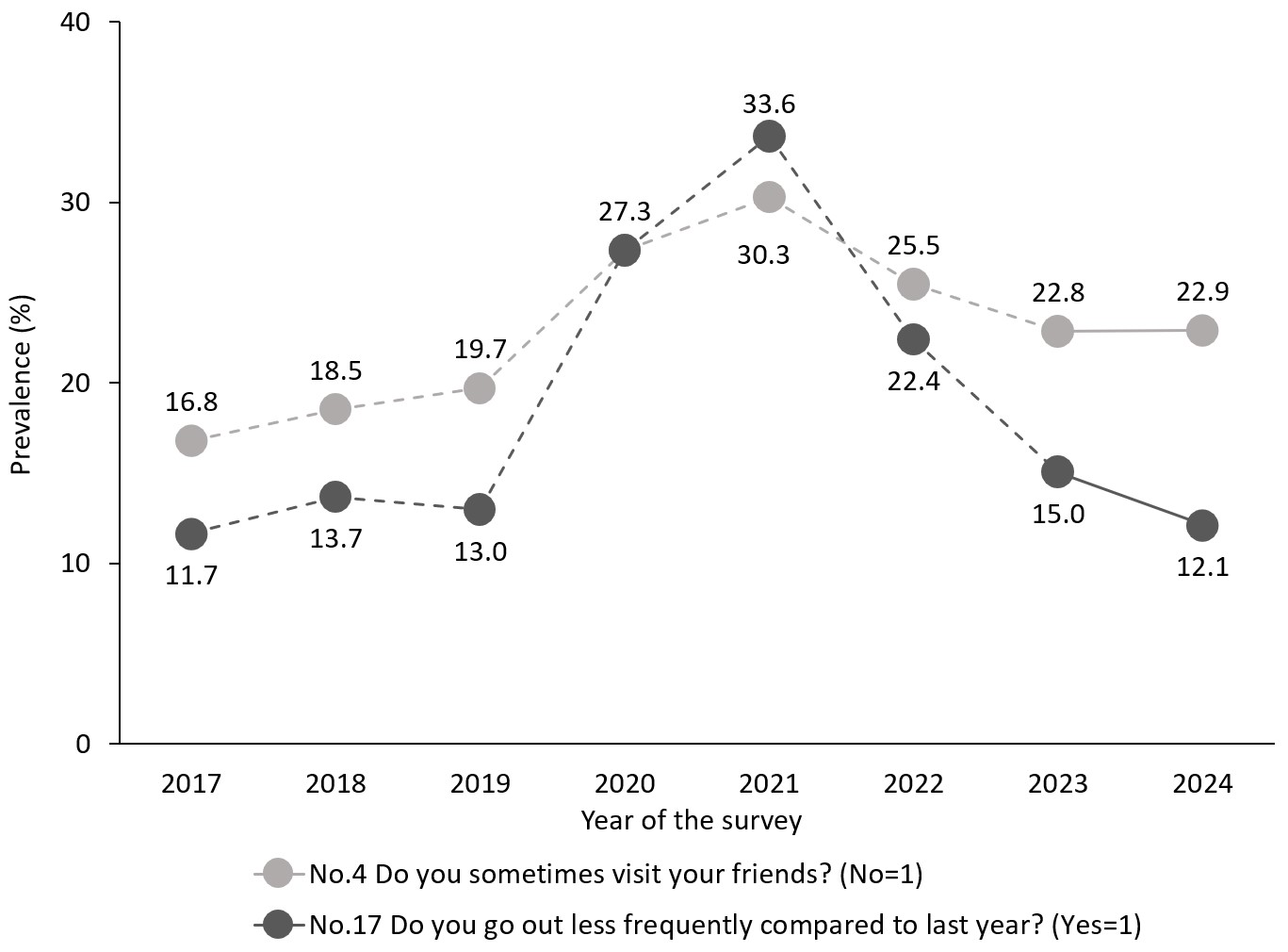


Figure S1. Changes in responses to No. 4 and 17 on the Kihon Checklist over 8 years.

By adding the 2024 analysis to our previously reported data, we present the proportion over 8 years of individuals who scored one point in the impaired direction on the KCL items: answering “No” to No. 4, “Do you sometimes visit your friends?” and “Yes” to No.17 “Do you go out less frequently compared to last year?” In 2024, the proportion of No. 17 returned to its pre-COVID-19 pandemic level, whereas the proportion of No. 4 remained comparable to that in 2023.
